# Out of focus: limited representation of men’s health needs in regional and global sexual and reproductive health policy

**DOI:** 10.1093/heapol/czaf090

**Published:** 2025-11-13

**Authors:** Tim Shand, Conor Evoy, Peter Baker, Dominick Shattuck, Morna Cornell, Derek M Griffith

**Affiliations:** ShandClarke Consulting, Queen Margaret University and Global Action on Men’s Health, Filsham Road, St Leonards-on-sea, TN38 0PA, United Kingdom; ShandClarke Consulting, Filsham Road, St Leonards-on-sea, TN38 0PA, United Kingdom; Global Action on Men’s Health, City Road, London, EC1V 2NX, United Kingdom; School of Medicine, and Bloomberg School of Public Health, Johns Hopkins University, Baltimore, MD 21218, United States; Centre for Integrated Data and Epidemiological Research (CIDER), School of Public Health, University of Cape Town, Rondebosch, Cape Town, 7700, South Africa; Global Action on Men’s Health, City Road, London, EC1V 2NX, United Kingdom; School of Nursing and the Perelman School of Medicine, University of Pennsylvania, Philadelphia, PA 19104, United States

**Keywords:** men’s health, sexual and reproductive health, policy analysis, gender and health, health equity, gender equality

## Abstract

Addressing men's own specific health concerns in the context of sexual and reproductive health (SRH) remains a largely neglected topic, despite growing levels of unmet SRH needs among men and the broader benefits of engaging men in the SRH of women and others. A comprehensive policy analysis explored how men are currently addressed and characterized within 37 key global and regional SRH-focused policies. Men's own SRH was found to be a significantly neglected policy issue. Less than half (43%) of the policies provided reference to men's SRH, and only 16% purposefully outlined steps to address men's own SRH needs. This contrasted with 78% of policies addressing women's SRH. Policies rarely provided sex disaggregated data nor targets on men's SRH outcomes. The inclusion of men was typically for solely instrumental reasons—in order to improve women's SRH. Men's SRH was best addressed within language on HIV and sexuality transmitted infections (STIs), particularly for men who have sex with men. Policy coverage was poor on men's SRH needs and roles in relation to contraception, fertility, sexual dysfunction, reproductive cancers, sexual pleasure, healthy relationships. and SRH-related discrimination. Only a quarter (24%) of the policies included a focus on one or more vulnerable male sub-group, with inadequate policy attention to the specific SRH needs of older men, disabled men, men living with serious health conditions, transgender people, and heterosexual men. An absence of focus on men's distinct SRH needs, alongside that of women, limits global understanding and visibility of SRH challenges particular to men, impeding the formulation of policies, programs, and funding priorities that sufficiently address men's needs. It also reinforces SRH as a women's sole burden and entrenches gender inequalities. Health policies should prioritize men's increased access to SRH information and care and better frame SRH as a critical part of men's lives.

Key messages

- Men’s own sexual and reproductive health (SRH) needs are a neglected policy issue: Only 16% of policies purposefully outlined steps to address men’s own SRH needs.
- Policies reinforce the assumption that gender in SRH only refers to populations of women.
- Policies do not address the broad range of topic areas relevant to men’s SRH needs nor sufficiently serve the SRH needs of different male sub-groups.
- Improving a focus on men’s SRH within policies can improve SRH for everyone and promote gender equality and women’s rights.

Men and boys, in their diversity, have critical and growing unmet needs across their sexual and reproductive health (SRH), which impact on their health and wellbeing. Global prevalence of sexuality transmitted infections (STIs) is increasing among men, who have higher levels of syphilis, chlamydia, gonorrhoea, and trichomoniasis than women across all age groups between 24 and 85 years (Fu et al. 2022). Men are less likely to test for HIV, know their HIV status, and access antiretroviral treatment (UNAIDS 2021), and have higher rates of AIDS-related mortality (UNAIDS 2023). Male infertility accounts for around 50% of infertility in couples (Leslie et al. 2023); however, men face poor quality and insufficient fertility services in many parts of the world (Leung et al. 2018). Male reproductive cancers, particularly prostate, testicular, and penile cancer, have climbed globally since 1980 and are now highly prevalent (Park et al. 2018). Prostate cancer is the third most common cancer globally, the incidence of which is expected to double by 2040 (Dall’Era 2023, James et al. 2024, Schafer et al. 2025). Male sexual dysfunction is increasing in prevalence globally, affecting both older and increasingly, younger men (Rastrelli and Maggi 2017, Li et al. 2022). Finally, male condoms and vasectomy account for only one-quarter of contraceptive use worldwide, a prevalence figure, which has not shifted since 1994 (Ross and Hardee 2017).

Advancing sexual and reproductive health is not a zero-sum issue. Men’s and women’s SRH are inextricably linked. Robust evidence shows that the meaningful engagement of men is important in improving women’s SRH (Ruane-McAteer et al. 2019) and in reducing intimate partner violence and improving couple relations (Doyle et al. 2023). The SRH of women and girls needs greater attention in policy and practice (Shand and Evoy 2024). Women and girls shoulder the majority of the reproductive health burden, particularly the consequences of pregnancy and childbirth (Shand and Marcell 2021). At the same time, SRH is frequently treated as solely a women’s domain and synonymous with the health needs of women and girls alone (Shand 2021). Despite the aforementioned unmet SRH needs among men, and the broader benefits of engaging men in the SRH of women and others, men’s own specific health needs in the context of SRH remains a largely neglected topic (Starrs et al. 2018). A critical knowledge gap in strengthening the focus on men’s own SRH needs is to understand and then seek to improve how men’s SRH is reflected in public policy. To contribute to understanding in this area, a recent comprehensive policy analysis explored how men are currently addressed and characterized within 37 key global and regional SRH focused policies, particularly in relation to HIV, STIs, contraception, fertility, sexual dysfunction, and reproductive cancers.

The analysis found that overall men’s specific SRH needs are a significantly neglected policy issue (see Fig. 1). Less than half of the policies analysed (43%) provided reference to men’s SRH (though this was often superficially, with the exception of male at-risk groups for HIV and STIs), and only 16% purposefully outlined steps to address men’s own SRH needs. This contrasted with 78% of policies addressing women’s SRH. Policies rarely provided sufficient sex disaggregated data that included men and women (only 21%), tending to include only data on women, and even fewer policies (14%) included specific targets on men’s SRH (Shand and Evoy 2024). No single policy provided a comprehensive set of data points on men’s SRH needs beyond a single SRH topic (such as only on STIs). This mirrors results of a global mental health policy analysis, which found that, despite men’s higher rates of suicide being well-known and publicized, only 48% of policies provided sex-disaggregated data (Leon and Colvin 2024a). Similar gaps in sex-disaggregated data have been found in policy on cancer (Leon and Colvin 2023) and primary health care (Leon and Colvin 2024b). Researchers on both men’s and women’s health have been highlighting the need for sex disaggregated data for some time (Hawkes and Buse 2013). An absence of male-specific data, alongside that of women, limits regional and global understanding and visibility of SRH challenges particular to men, impeding the formulation of policies, programmes, and funding priorities that sufficiently address men’s SRH needs.

**Figure 1.**
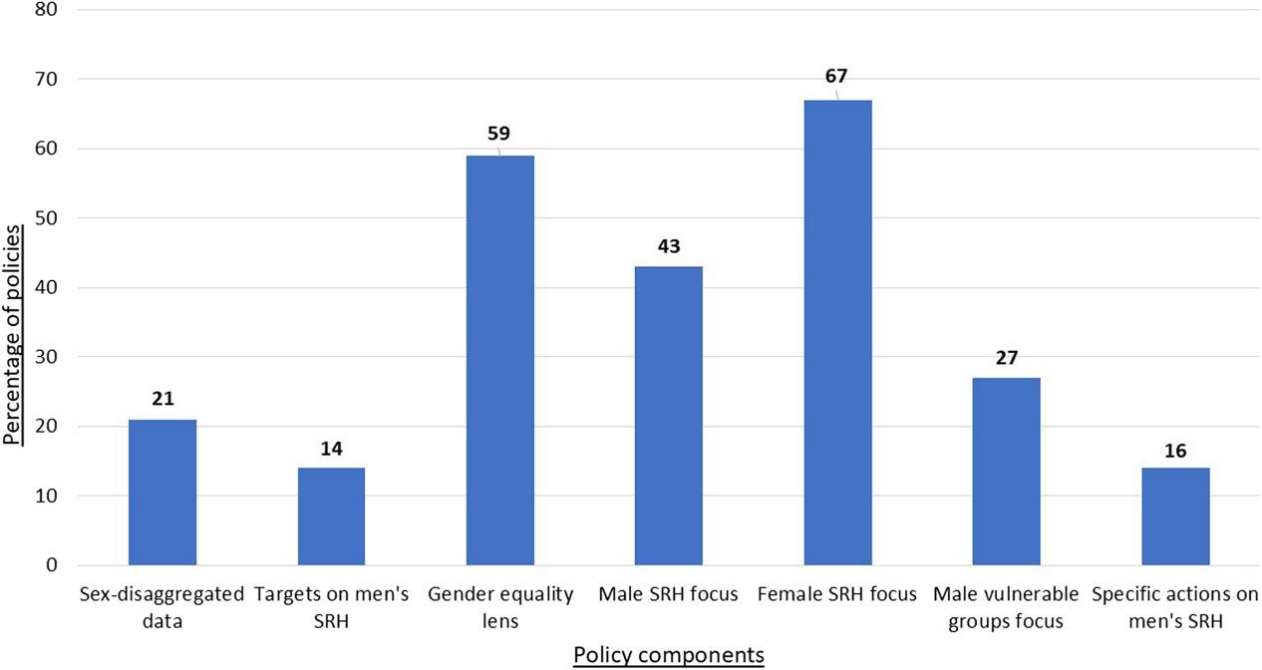
Overall inclusion of men’s SRH focus in global and regional SRH policies.

Policies reinforced the assumption that gender (the socially constructed characteristics of being a man or women) in SRH only refers to populations of women (Hawkes and Buse 2013), with men’s gendered behaviours and needs largely overlooked. This reflects how gender is often synonymous in global health policy and practice with women rather than as a relational construct that affects everyone’s health (Hawkes et al. 2025); an assumption that is reinforced by the UN and multilateral health agencies through their historical framing of gender in these narrow terms and their public commitments that equate gender equality with only the needs of women and girls (Hawkes et al. 2025). Men’s inclusion in health policies was found to be primarily in order to address (and ‘manage’) specific at-risk demographics—such as men who have sex with men (MSM)—or to explore the negative and harmful impact of male norms, roles or behaviours on women and others. Few policies sought to strategically engage men in challenging or shifting these norms. This reflected the almost ubiquitous inclusion of men in SRH policy for solely instrumental reasons—in order to improve women’s SRH outcomes. While it is critical for policy to acknowledge and tackle men’s harmful behaviours, the absence of focus on men’s distinct SRH needs—as compared to women’s—reinforces inequities in SRH care for men. The failure of SRH policy to integrate men in a meaningful way also reinforces traditional gender and patriarchal norms that define SRH as a domain that has no purpose for men or value in men being positively engaged, despite the fact that power differentials mean that men can restrict women’s access to SRH information and services. This reinforces SRH as a women’s sole burden and further entrenches gender inequalities. These missed opportunities to work with men on their broader health and wellbeing is also reflected in policies focused on cancer health care, where gender is referred to rarely and, where cited, it is typically focused on women’s specific needs (Leon and Colvin 2023). In fact, many men affected by cancer have unique physiological and psychological SRH concerns (e.g. sexual dysfunction) that are currently frequently overlooked (Hung et al. 2020, Rogers et al. 2022, Dickstein et al. 2023).

Additionally, analysed policies failed to address the broad range of topics relevant to men’s SRH needs (see Fig. 2). The most comprehensive policy coverage is on STIs and HIV, which contain language about prevention, treatment, and care for men. However, these policies tend to focus mostly on high-risk groups such as MSM and transgender individuals, overlooking cisgender heterosexual men and older men. Opportunities to improve men’s understanding and participation in contraceptive services are underrepresented in policy, with the majority of policies (87%) framing contraception as solely a women’s SRH issue. Even fewer policies contain a dedicated focus on men as contraceptive users. Male infertility is only addressed in 16% of policies, with content generally focused on female infertility. Sexual dysfunction, including erectile dysfunction and premature ejaculation, is almost entirely absent in policies, with only 5% acknowledging this issue, despite its rising prevalence globally. References to men’s reproductive cancers are similarly rare (5% of policies), in comparison to the important body of policy dedicated to cervical cancer prevention and treatment. Positive aspects of SRH, such as sexual pleasure and healthy relationships are insufficiently covered for both men and women. Discrimination that some men experience related to access to SRH care due to racism or disability are poorly addressed. Lastly, most policies importantly focus on men as perpetrators of violence against women; yet, only one-fifth (19%) acknowledge men’s experience of violence, and these references are typically brief (Shand and Evoy 2024).

**Figure 2.**
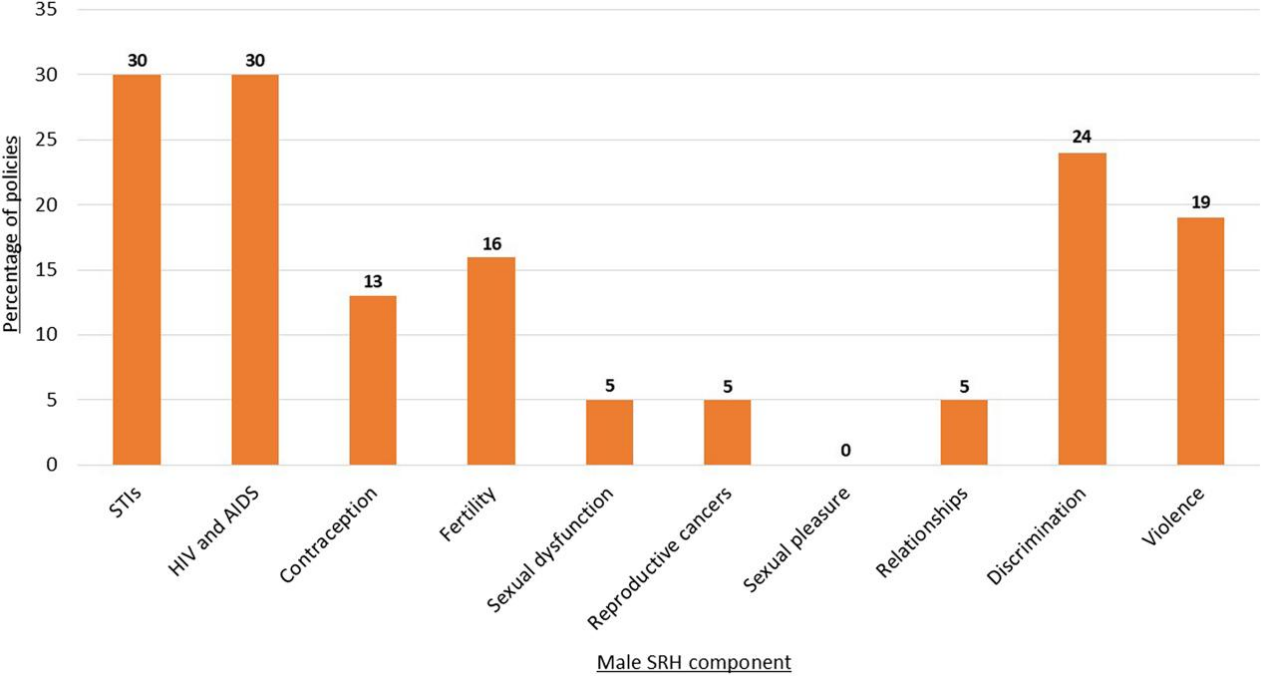
Coverage of male SRH topics in SRH policies.

Policy also poorly serves the SRH of different sub-groups of men (see Fig. 3) with only a quarter (24%) including a focus on one or more vulnerable male group. Young men’s SRH needs are rarely addressed, instead often being referred to in gender neutral terms as ‘young people’ or grouped into ‘men and boys’, without further reference to their unique needs. Older men are severely underrepresented in SRH policy, with only 5% of policies making any reference to them. Disabled men are absent from mainstream SRH policies, with no single meaningful reference to their needs. Men living with serious health conditions, such as reproductive cancers, are similarly overlooked. MSMs are one of the most well-represented groups within SRH policy, particularly in the context of HIV and STIs; however, there is little attention given to other aspects of their SRH, such as reproductive cancers or fertility. Transgender people are rarely mentioned in SRH policies, and, as with MSM, when they are, it is almost always in the context of HIV and STIs only. Transgender people includes both *trans* women who have male biological SRH needs (such as being likely to have a prostate) and *trans* men who may have SRH needs (such as sexual dysfunction) if they go through medical transition, though policy rarely distinguishes between these two groups. Finally, the needs of heterosexual men are largely ignored, with only 3% of the policies including a deliberate focus on this group, despite the significant and growing SRH concerns among heterosexual men (Shand and Evoy 2024). Addressing the SRH needs of all these groups is important as part of advancing a more inclusive definition of men’s health (White et al. 2023).

**Figure 3.**
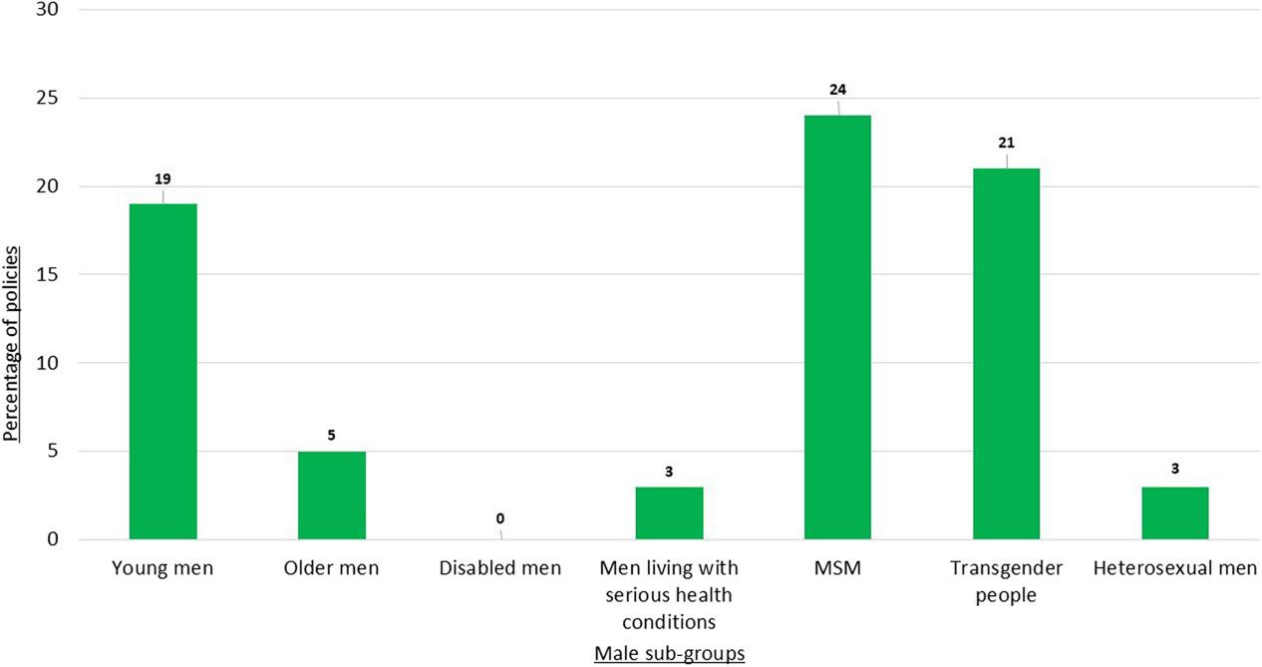
Representation of male sub-groups in SRH policies.

These findings highlight the persistent neglect of men’s health in policies on SRH. Similar findings have been documented in other health policy areas, such as tuberculosis (Cornell et al. 2020). This can be attributed, in part, to the reluctance of some donors, programme managers, and SRH advocates to promote availability of SRH services for men (Edstrom et al. 2015) and that some may view investing in men’s SRH needs as taking away funds from women’s needs (Starrs et al. 2018). A body of interventions has now focused on engaging men and promoting alternative masculine norms in the context of SRH (Ruane-McAteer et al. 2019, Ruane-McAteer et al. 2020). Important those these are, these gender-transformative interventions reinforce the aforementioned instrumental engagement of men and few programmes on masculinities and SRH attend to the pathways through which gender ideals affect men’s own health needs. The findings of this analysis also point to how the results of these gender-transformative interventions have also not been reflected in global SRH policy (the focus of this analysis), mirroring findings that such programmes are not scaled up after the pilot stage (Starrs et al. 2018). While gender-transformation is an essential programming approach, it is unclear at best what policy goals should seek to change or transform in order to address men’s sexual dysfunction, infertility, and reproductive cancers (Fisher and Makleff 2022).

Global health policies urgently need to prioritize men’s increased access to SRH information, services, and care, as well as frame SRH as a critical part of men’s lives. These efforts should be coupled with expanding data collection and targets on men’s SRH needs. Policies should be revised to more actively engage men in challenging harmful gender norms and as allies in women’s and children’s SRH. At a time of global pushback on advances in sexual and reproductive health and rights and cuts to budgets for SRH programming, policies should assert that men can, and should, play a key role in speaking out on the importance of everyone’s SRH. Policies should further include a focus on the broad range of male SRH topics, particularly, the more neglected areas of infertility, sexual dysfunction, and reproductive cancers. And policies should give greater attention to the needs of different male sub-groups to ensure more equitable and inclusive SRH responses. Doing so will not only make men’s SRH more visible and improve their SRH outcomes, but will facilitate better engagement of men as supportive partners and agents of change in the SRH of women, other men and non-binary individuals and in improving gender equality (Gottert et al. 2025).

## Data Availability

The data underlying this article will be shared on reasonable request to the corresponding author.

## Reflexivity statement

The authors include five males and one female and span multiple levels of seniority, three geographical regions and different sectors. The authorship team includes academics, civil society actors, and a small consultancy firm, and this work was led by the civil society actors. One author is based in the global South. One author is at an early stage in their career. With the exception of the early-stage career author, all others have worked for several decades on men’s health, including men’s sexual and reproductive health, including in both global North and global South contexts. We acknowledge that the authorship team is predominantly based in high-income countries, though this also reflects where the civil society actors and consultancy firm are based and the research was global in nature.
